# Fractional optimal control strategies for hepatitis B virus infection with cost-effectiveness analysis

**DOI:** 10.1038/s41598-023-46849-8

**Published:** 2023-11-09

**Authors:** Lemesa Bedjisa Dano, Purnachandra Rao Koya, Temesgen Duressa Keno

**Affiliations:** Department of Mathematics, Wollega University, Nekemte, Ethiopia

**Keywords:** Cell biology, Computational biology and bioinformatics

## Abstract

Hepatitis B disease is a communicable disease that is caused by the hepatitis B virus and has become a significant health problem in the world. It is a contagious disease that is transmittable from person to person either horizontally or vertically. This current study is aimed at sensitivity analysis and optimal control strategies for a fractional hepatitis B epidemic model with a saturated incidence rate in the sense of the Caputo order fractional derivative approach. Fundamental properties of the proposed fractional order model are obtained and discussed. A detailed analysis of disease-free equilibrium and endemic equilibrium points is given by applying fractional calculus theory, which is a generalized version of classical calculus. Sensitivity indexes are calculated for the classical order model. Illustrative graphics that show the dependence of the sensitivity index on fractional order derivative for $\alpha \in (0,1)$ are provided. Based on the results of the sensitivity analysis and using Pontryagin’s Maximum Principle, optimal control strategies for preventing hepatitis B infection with vaccination and treatment are considered. Fractional Euler’s method is used to carry out the numerical simulation for the proposed fractional optimal control system and the obtained results are analyzed. The results of the analysis reveal that hepatitis B disease can be prevented if necessary precautionary is taken or effective vaccination and treatment control measures are applied. The analysis of cost-effectiveness is also conducted and discussed.

## Introduction

Hepatitis B infection is a contagious liver illness caused by the hepatitis B virus (HBV). This mostly targets the liver organ in the human body, causing persistent infection and eventually progressing to cirrhosis or liver cancer, putting people at high risk of mortality. The main causes of hepatitis infection are bacterial or viral infections, as well as active exposure to alcohol and drugs. HBV infection is a worldwide health issue that causes both acute and chronic infections and can progress to cirrhosis^1^. Acute HBV infection occurs within the first 6 months after exposure. During this time, the immune system may be able to clear the HBV from the diseased body, but it may also progress to the serious stage in certain extreme cases, resulting in lifelong illness. Chronic liver disease affects over 2 billion people worldwide, with around 400 million people living with chronic hepatitis B and C^2^. Every year, an estimated 1 million individuals die as a result of the acute or chronic consequences of hepatitis B. As a result, quick action and robust measures are required to minimize the disease burden on our health system and reduce disease-related mortality. The various stages of hepatitis B infection, including both vertical and horizontal spread, are critical in the transmission of hepatitis B infection. Chronic carriers, in particular, are highly important since they have no symptoms yet nevertheless transmit the disease (see^3,4^ and refs therein).

In epidemiology, mathematical modeling of infectious disease plays an important role in investigating disease dynamics and suggesting disease control mechanisms; as a result, a vast body of epidemiological model works on hepatitis B disease have been developed^5–11^. In addition, the theory of optimal control in epidemiology plays a significant role that provides appropriate preventive control strategies. Khan et al.^6^ studied the effect of migration, hospitalization, and treatment rate for the hepatitis B epidemic model qualitatively. Din et al.^7^ applied vaccination and treatment as control measures on a SABR hepatitis B model to control the spread of the disease. Ullah et al.^8^ investigated the effect of vaccination and treatment on the hepatitis B model to control the spread of HBV infection. The authors in^9^ developed a deterministic model to study the dynamics of HBV infection. In^10^ the merits of media coverage on the transmission of the hepatitis B epidemic model were presented.

The above mathematical models are classical order models and do not consider the past state of those infectious diseases. In the last decades, the idea of fractional calculus is widely used to study the dynamics of real-world problems in various branches of sciences^2,12–23^. Fractional calculus is characterized by non-locality and non-singularity kernels. So, many authors used different definitions for fractional derivatives including the Caputo derivative, Caputo-Fabrizio (CF) derivative, and Atangana–Baleanu (AB) derivatives extensively to model various infectious diseases. This is because the fractional-order derivative captures memory effects and hereditary properties, and it is the generalization of the integer-order derivative, which is local. Due to the non-locality behavior in fractional order models, various choices exist in the order of the derivatives. They can accurately explain natural phenomena better than classical ones, thus, fractional calculus has taken on the popularity of modeling realistic situations, especially those with memory effects.

Ullah et al.^12^ developed a new HBV model with hospitalization via Caputo fractional-order derivative. The authors in^13^ studied the dynamics of fractional HBV with Holling type II functional response to investigate the virus-to-cell transmission of the disease. Khan et al.^14^ formulated a deterministic SIR hepatitis B virus infection using an epidemic model with three time-dependent control variables. Simalene and Dlamini^15^ extended the work in^14^ using fractional order differential equations via Caputo-type fractional order derivative in the absence of any control measure. In^17^, the authors studied the asymptomatic carriers class of hepatitis B infection via the Caputo-fractional derivative. In^18^, the authors investigated the dynamics of the fractional hepatitis B model with the impact of hospitalization of acute and chronically infected individuals. In^19^, the authors studied chronic HBV infection which rapidly progresses to liver cirrhosis leading to frequent hospitalization using fractional order differential equations. In^22^, the authors developed HBV epidemic model with two age structures in the presence of vaccination. Ullah et al.^24^ and Liu et al.^25^ formulated fractional order modeling of Nipah virus and monkeypox virus infections, respectively to investigate the dynamics of the diseases using Caputo fractional derivative. The authors in^26^ studied the dynamics of HBV via Atangana–Baleanu fractional operators to investigate the non-singularity and non-locality of the model.

However, none of the above scholars considered the fractional optimal control problem of the SIR hepatitis B epidemic model with a saturated incidence rate. In this paper, we will extend the work in^15^ to a fractional optimal control problem by considering two time-dependent control variables. These control measures are used to explore the impact of vaccination on susceptible individuals to protect them from severe infection and treatment of infected individuals. This new assumption may be more useful in eliminating the disease and suggest deeper insights for studying its dynamics.

## Preliminaries

Fractional order derivatives and fractional integrals are the generalizations of ordinary derivatives and integrals. Here, we present some basic definitions, and important lemmas and theorems on fractional calculus.

### Definition 1

(^27^) Suppose $z :[a,b] \rightarrow {\mathbb {R}}$ is a function with $\alpha \in (0,1]$. Thus, the left and right Riemann–Liouville fractional integrals, respectively are defined as follows

$$\begin{aligned} {_{a}}{I_{t}^{\alpha }}{z(t)} = \frac{1}{\Gamma (\alpha )}{\int _{a}^{t}(t-s)^{\alpha -1}z(s)ds} \end{aligned}$$

and

$$\begin{aligned} {_{t}}{I_{b}^{\alpha }}{z(t)} = \frac{1}{\Gamma (\alpha )}{\int _{t}^{b}(s- t)^{\alpha -1}z(s)ds}, \end{aligned}$$

where $a< t \le b < \infty$ and $\Gamma (\cdot )$ is the Euler’s gamma function.

### Definition 2

(^28^) Suppose $z :[a,b] \rightarrow {\mathbb {R}}$ is a function with $\alpha \in (0,1]$. Thus, the left and right Riemann–Liouville derivatives of order $\alpha > 0$, respectively are defined as follows

$$\begin{aligned} {_{a}^{RL}}{D_{t}^{\alpha }}{z(t)} =\frac{d^{n}}{dt^{n}}({{_{a}}{I_{t}^{n-\alpha }}{z(t)}})= \frac{1}{\Gamma (n-\alpha )} \left( \frac{d}{dt} \right) ^{n} {\int _{a}^{t}(t-s)^{n-\alpha -1}z(s)ds} \end{aligned}$$

and

$$\begin{aligned} {_{t}^{RL}}{D_{b}^{\alpha }}{z(t)} = \frac{d^{n}}{dt^{n}} ({{_{a}}{I_{t}^{n-\alpha }}{z(t)}})= \frac{(-1)^{n}}{\Gamma (n-\alpha )} \left( \frac{d}{dt} \right) ^{n} {\int _{t}^{b}(s-t)^{n-\alpha -1}z(s)ds}. \end{aligned}$$

### Definition 3

(^27,28^) Suppose $z :C^{n}[a,b] \rightarrow {\mathbb {R}}$ is a function with $n-1 < \alpha \le n,~n \in {\mathbb {Z}}$. Thus, the left and right Caputo fractional derivatives of order $\alpha > 0$, respectively are defined as follows

$$\begin{aligned} {_{a}^{C} D_{t}^{\alpha }}{z(t)} = \frac{1}{\Gamma (n-\alpha )} {\int _{a}^{t}{(t-s)^{n-\alpha -1}z^{(n)}(s)ds}}, \end{aligned}$$

and

$$\begin{aligned} {_{t}^{C} D_{b}^{\alpha }}{z(t)} = \frac{1}{\Gamma (n-\alpha )} {\int _{t}^{b}{(s-t)^{n-\alpha -1}z^{(n)}(s)ds}}. \end{aligned}$$

### Definition 4

(^27^) The fixed point $z^{*}(t)$ represents the solution of the Caputo fractional order derivative of the form

$$\begin{aligned} {_{0}^{C}{D_{t}^{\alpha }}{z(t)}} = f(t,z(t)), ~ ~ 0< \alpha <1 \end{aligned}$$

if and only if $f(t,z^{*}(t))=0,~\forall {t \in [0,b]}$.

Since the local asymptotical stability of the fractional order system is quite different from the ordinary integer order model, we need to define the stability of the model according to the next theorem.

### Theorem 1

*For* $\alpha \in (0,1]$ *and* $z :{\mathbb {R}}^{+} \times {\mathbb {R}}^{n} \rightarrow {\mathbb {R}}^{n}$. *Consider the fractional order system of the form*

$$\begin{aligned} \begin{aligned}{}&{^{C}}{D_{t}^{\alpha }}{z(t)} = f(t,z(t)),\\&z(0) = z_{0}. \end{aligned} \end{aligned}$$

*If all eigenvalues* $\lambda _{i} ~(i=1,\dots ,n)$ *of the jacobian matrix* $\frac{\partial f}{\partial z}$ *calculated at the equilibrium points of the fractional order system satisfy* $\big |arg(\lambda _{i}) \big | > \frac{\alpha \pi }{2}$, *then the equilibrium points are locally asymptotically stable.*

### Theorem 2

*Suppose* $z :[0, b] \rightarrow {\mathbb {R}}$ *is a function with* $n-1<\alpha \le n, ~n \in {\mathbb {N}}$ *for all* $t>0$. *The following relation between Riemann–Liouville and Caputo fractional derivatives always hold*

$$\begin{aligned} {_{0}^{RL}}{D_{t}^{\alpha }}{z(t)} = {_{0}^{C}}{D_{t}^{\alpha }}{z(t)}+\sum _{k=0}^{n-1}{\frac{z^{(k)}(0)}{\Gamma (k-\alpha +1)} {t}^{(k-\alpha )}}, \end{aligned}$$

*and*

$$\begin{aligned} {_{t}^{RL}}{D_{b}^{\alpha }}{z(t)} = {_{t}^{C}}{D_{b}^{\alpha }}{z(t)}+\sum _{k=0}^{n-1}{\frac{z^{(k)}(b)}{\Gamma (k-\alpha +1)} (b-t)^{(k-\alpha )}}. \end{aligned}$$

*Thus, if* $z(0) = z'(0) = \dots = z^{(n-1)}(0) = 0$, *then* ${_{0}^{RL}}{D_{t}^{\alpha }}{z(t)} = {_{0}^{C}}{D_{t}^{\alpha }}{z(t)}$ and if $z(b) = z'(b) = \dots = z^{(n-1)}(b) = 0$, *then* ${_{t}^{RL}}{D_{b}^{\alpha }}{z(t)} = {_{t}^{C}}{D_{b}^{\alpha }}{z(t)}$.

### Definition 5

(^29^) For $z \in {\mathbb {C}}$, the one parameter and two parameters Mittag–Leffler functions with $\alpha , \beta \ge 0$ is defined by

$$\begin{aligned} \begin{aligned}{}&E_{\alpha , 1}{(z)} = E_{\alpha }{(z)} = \sum _{k=0}^{\infty }{\frac{z^k}{\Gamma (\alpha k+1)}}~~\text {and}\\&E_{\alpha , \beta }{(z)} = \sum _{k=0}^{\infty }{\frac{z^k}{\Gamma (\alpha k+\beta )}} \end{aligned} \end{aligned}$$

and satisfies the property

$$\begin{aligned} E_{{\alpha },{\beta }}(z) = z E_{{\alpha },{\alpha +\beta }} (z) + \frac{1}{\Gamma (\beta )}. \end{aligned}$$

### Theorem 3

*The Laplace transform of the Caputo derivative operator of order* $\alpha > 0$ *of the function* *z*(*t*) *is defined as*

$$\begin{aligned} {\mathscr {L}} \big [{_{0}^{C}}{D_{t}^{\alpha }}{z(t)};s \big ] = s^{\alpha }Z(s)-\sum _{k=0}^{n-1}{z^{k}(0)s^{\alpha -k-1}}. \end{aligned}$$

### Definition 6

The Laplace transform of the Mittag–Leffler function of the form $t^{\beta -1}E_{{\alpha },{\beta }}(\pm \lambda t^{\alpha })$ is defined as

$$\begin{aligned} {\mathscr {L}} [t^{\beta -1}E_{{\alpha },{\beta }}(\pm \lambda t^{\alpha });s] = \frac{ s^{\alpha -\beta }}{s^{\alpha }\mp \lambda }. \end{aligned}$$

### Lemma 1

(^21^) *Assume that* $z :[0,T] \rightarrow {\mathbb {R}}$ *and* $0 < \alpha \le 1$. *Thus,*

$$\begin{aligned} {_{t}^{C}D_{T}^{\alpha }z(t)} = {_{0}^{C}D_{t}^{\alpha }z(T-t)}. \end{aligned}$$

## Mathematical model

Based on the work of Simalene and Dlamini^15^, we extended the fractional order modeling for the hepatitis B virus infection with saturated incidence rate to fractional optimal control problem using the Caputo fractional order derivative. According to the model, the total population *T*(*t*) is divided into three sub-classes; namely susceptible, infected, and recovered individuals. The SIR model is the simplest and most common model for describing and predicting the epidemics of any contagious disease (see^30^ and refs therein). Thus, the total population is given by $T(t)=S(t)+I(t)+R(t)$.

Simalene and Dlamini^15^ fractionalised hepatitis B epidemic model in the work of Khan et al.^14^ presented in the Mathematical Model Formulation section using Caputo fractional order derivative.

1 $$\begin{aligned} {} {\left\{ \begin{array}{ll} {_{0}}{D_{t}^{\alpha }}{S(t)} = \Lambda -\frac{\alpha S(t) I(t)}{1+\gamma I(t)}-(\mu _{0} +\nu )S(t),\\ {_{0}}{D_{t}^{\alpha }}{I(t)} = \frac{\alpha S(t) I(t)}{1+\gamma I(t)}-( \mu _{0}+\mu _{1} +\beta )I(t),\\ {_{0}}{D_{t}^{\alpha }}{R(t)} = \beta I(t)+\nu S(t)-\mu _{0} R(t). \end{array}\right. } \end{aligned}$$

However, we remark that the fractional order system (1) does not have appropriate dimensions. Indeed, the left-hand time dimension is $\textit{(}time)^{- \alpha }$, whereas the right-hand time dimension is $\textit{(}time)^{-1}$. That is, the fractional-order system (1) is only consistent when the order of differentiation $\alpha = 1$. We translated the dynamics of HBV in each population class using Caputo-fractional derivatives of order $\alpha \in (0,1]$. As a result, the fractional order model (FOM) described in Eq. (2) captures memory effects, which are critical for accurately defining the bio-dynamic model. As a result, the primary goal of studying fractional order derivatives is to gain a better understanding of disease dynamics. Hence, the fractional order problem characterizes the HBV spreading dynamics using the Caputo fractional derivative.

2 $$\begin{aligned} {} {\left\{ \begin{array}{ll} {_{a}^{C}}{D_{t}^{\alpha }}{S(t)} = \Lambda ^{\alpha }-\frac{\beta ^{\alpha } S(t) I(t)}{1+\gamma ^{\alpha } I(t)}-(\mu _{0}^{\alpha }+\nu ^{\alpha })S(t),\\ {_{a}^{C}}{D_{t}^{\alpha }}{I(t)} = \frac{\beta ^{\alpha } S(t) I(t)}{1+\gamma ^{\alpha } I(t)}-(\mu _{0}^{\alpha }+\mu _{1}^{\alpha } +\sigma ^{\alpha })I(t),\\ {_{a}^{C}}{D_{t}^{\alpha }}{R(t)} = \sigma ^{\alpha } I(t)+\nu ^{\alpha } S(t)-\mu _{0}^{\alpha } R(t). \end{array}\right. } \end{aligned}$$

where ${_{a}^{C}}{D_{t}^{\alpha }}$ represents the left Caputo fractional-order derivative with derivative order $\alpha \in (0.1]$ along with initial conditions

3 $$\begin{aligned} {} S(0) = S_{0} \ge 0, I(0) = I_{0} \ge 0, R(0) = R_{0} \ge 0 \end{aligned}$$

Table 1 describes the biological description, parameter’s value, and their sources.

**Table 1:** Biological description of model parameters of FOM (2), and parameter’s value with their corresponding sources.

Parameter	Description	Value	Unit	Source
$\Lambda$	Birth rate	0.2320000	Per day	^15^
$\beta$	Transmission rate	0.0009000	Per day	^15^
$\gamma$	Saturation rate	0.9000000	Per day	^14,15^
$\mu _{0}$	Natural death rate	0.0002320	Per day	^15^
$\nu$	Vaccination rate	0.0200000	Per day	^14,15^
$\mu _{1}$	Induced death rate	0.0000547	Per day	^14^
$\sigma$	Recovery rate	0.1200000	Per day	^15^

In FOM (2), the quantity $\frac{\beta ^{\alpha }S(t) I(t)}{1+\gamma ^{\alpha } I(t)}$ represents the saturated incidence rate in which the factor $\frac{\beta ^{\alpha }I(t)}{1+\gamma ^{\alpha } I(t)}$ shows the saturation level reached whenever *I*(*t*) increases, $\beta ^{\alpha } I(t)$ measures the infection force when the disease is entering a totally susceptible population, and $\frac{1}{1+\gamma ^{\alpha }I(t)}$ measures the inhibition effect from the behavioral change of susceptible individuals when their number increases. This incidence rate is more reasonable than the bilinear incidence rate since it accounts for behavioral change and prevents the contact rate from becoming unbounded by choosing suitable parameters. In this model, we also assumed that the functions *S*(*t*), *I*(*t*), *R*(*t*) and their Caputo fractional derivatives are continuous for all $t \ge 0$.

## Mathematical analysis

Let’s denote ${\mathbb {R}}_{+}^{3} = \{z(t) \in {\mathbb {R}}^{3} :z(t) \ge 0\}$ and let $z(t)=(S(t),I(t),R(t))^{T}$. Next, we investigate the non-negativity and boundedness of the FOM (2) by stating the following lemma to prove the result about the non-negativity and boundedness.

### Lemma 2

(^31^, Generalized Mean Value Theorem) *Assume that* $z(t) \in C[a,b]$ *and let* $_{a}^{C}{D_{t}^{\alpha }}z(t) \in C[a,b]$ *for* $\alpha \in (0,1]$. *Thus, we have*

$$\begin{aligned} z(t) = z(a)+\frac{1}{\Gamma (\alpha )}{_{a}^{C}{D_{t}^{\alpha }}z(\zeta )}{(t- a)^{\alpha }}, \end{aligned}$$

*where* $$0 \le \zeta \le t$$ *for* $$a \le t \le b$$.

From Lemma 2, we make the following remark.

### Remark 1

Suppose that $z(t) \in C[a,b]~\text {and}~{_{a}^{C}D_{t}^{\alpha }} \in C[a,b]$ for $0 < \alpha \le 1$. Thus, we have

1. If ${_{a}^{C}D_{t}^{\alpha } z(t)} \ge 0 ~ \forall {t} \in [a,b]$, then the function *z*(*t*) is non-decreasing for each $t \in [a,b]$. and
2. If ${_{a}^{C}D_{t}^{\alpha } z(t)} \le 0 ~\forall {t} \in [a,b]$, then the function *z*(*t*) is non-increasing for each $t \in [a,b]$.

### Theorem 4

*The solution* (*S*(*t*), *I*(*t*), *R*(*t*)) *of FOM* (2) *are non-negative if the initial data* (*S*(0), *I*(0), *R*(0)) *are non-negative for* $\alpha \in (0,1], ~\forall {t}>0$.

### Proof

Now, we prove that the region $\Omega$ is the positivity invariant set for FOM (2). Hence,

4 $$\begin{aligned} {} {\left\{ \begin{array}{ll} {_{a}^{C}}{D_{t}^{\alpha }}{S(t)}|_{S=0} = \Lambda ^{\alpha }>0,\\ {_{a}^{C}}{D_{t}^{\alpha }}{I(t)}|_{I=0}>0,\\ {_{a}^{C}}{D_{t}^{\alpha }}{R(t)}|_{R=0} >0. \end{array}\right. } \end{aligned}$$

Since $(S(0), I(0), R(0)) \in {\mathbb {R}}_{+}^{3}$ and all parameters in the FOM (2) are positive, then from *Lemma*  2, the solution (*S*(*t*), *I*(*t*), *R*(*t*)) remain in ${\mathbb {R}}_{+}^{3}$. Hence, the region $\Omega$ is the positivity invariant set for FOM (2). $\square$

### Theorem 5

*The closed region* $\Omega = \bigg \{(S(t),I(t),R(t)): S(t)\ge 0, I(t)\ge 0, R(t)\ge 0, ~T(t) \le \left( \frac{\Lambda }{\mu _{0}} \right) ^{\alpha } \bigg \}$ *is a positive invariant set in the FOM* (2) *for* $\alpha \in (0,1]$.

### Proof

Upon adding equations of FOM (2) by taking account that $S+I+R=T$, we obtain

5 $$\begin{aligned} {} {_{0}^{C}D_{t}^{\alpha }T(t)} = \Lambda ^{\alpha }-\mu _{0}^{\alpha }T(t)-\mu _{1}^{\alpha } I(t) \le \Lambda ^{\alpha }-\mu _{0}^{\alpha }T(t). \end{aligned}$$

Applying the Laplace transform on Eq. (5) using Theorem 3, we obtain

6 $$\begin{aligned} {} s^{\alpha }{T(s)}- s^{\alpha -1}T(0) \le \frac{\Lambda ^{\alpha }}{s} \mu _{0}^{\alpha }{T(s)}. \end{aligned}$$

Further, the simplification of Eq. (6) yields

7 $$\begin{aligned} {} {T(s)} \le \frac{s^{-1}}{s^{\alpha }+\mu _{0}^{\alpha }}\Lambda ^{\alpha }+ \frac{s^{\alpha -1}}{s^{\alpha }+\mu _{0}^{\alpha }}T(0). \end{aligned}$$

From Eq. (7) and using $(S(0),I(0),R(0)) \in \Omega$, we conclude that

$$\begin{aligned} \begin{aligned} T(t)&\le \Lambda ^{\alpha } t^{\alpha } E_{{\alpha },{\alpha +1}}(-\mu _{0}^{\alpha } t^{\alpha }) + E_{{\alpha },{1}}(-\mu _{0}^{\alpha } t^{\alpha }) T(0)\\&\le \left( \frac{\Lambda }{\mu _{0}} \right) ^{\alpha } \left[ E_{{\alpha },{\alpha +1}}(-\mu _{0}^{\alpha } t^{\alpha }) + E_{{\alpha },{1}}(-\mu _{0}^{\alpha } t^{\alpha }) \right] = \left( \frac{\Lambda }{\mu _{0}} \right) ^{\alpha }. \end{aligned} \end{aligned}$$

Clearly, we obtain the boundedness of *T*(*t*) as $0<T(t) \le \left( \frac{\Lambda }{\mu _{0}} \right) ^{\alpha }$. Hence, the feasible region $\Omega$ is positively invariant. This shows that the solution of the model is bounded. Therefore, FOM (2) is both mathematically well-posed and epidemiological meaningful. $\square$

### Equilibria and stability analysis

Basically, FOM (2) has two significant equilibrium points namely: the disease-free-equilibrium (DFE) and endemic equilibrium (EE) points. Setting the right-hand side of Eq. (2) to zero in the absence of no infectives will give us the DFE of FOM (2). Let ${\mathscr {E}}_{0}=\left( S(0),0,0 \right)$ denote DFE, where $S(0) = \frac{\Lambda ^{\alpha }}{\mu _{0}^{\alpha }+\nu ^{\alpha }}$. To examine the local stability of the equilibria points, one needs to calculate the basic reproduction number by the next-generation matrix approach. Here, the matrices *F* and *V* at ${\mathscr {E}}_{0} = \left( \frac{\Lambda ^{\alpha }}{\mu _{0}^{\alpha }+\nu ^{\alpha }},0,0 \right)$ are calculated as

$$\begin{aligned} F = \bigg [\frac{\partial {\mathscr {F}}_{i} ({\mathscr {E}}_{0})}{\partial z_{i}} \bigg ] = \beta ^{\alpha } S_{0} ~~~~ \text {and} ~~~~ V = \bigg [\frac{\partial {\mathscr {V}}_{i} ({\mathscr {E}}_{0})}{\partial z_{i}} \bigg ] = \left( \mu _{0}^{\alpha }+\mu _{1}^{\alpha }+\sigma ^{\alpha } \right) , \end{aligned}$$

where *F* is the non-negative matrix for new infections terms and *V* is the non-singular matrix for the remaining transition terms. Now, the spectral radius $(\rho (F V^{-1}))$ is calculated as

$$\begin{aligned} \rho (FV^{-1}) = \frac{\Lambda ^{\alpha } \beta ^{\alpha }}{(\mu _{0}^{\alpha }+\nu ^{\alpha })(\mu _{0}^{\alpha }+\mu _{1}^{\alpha }+\sigma ^{\alpha })}. \end{aligned}$$

Therefore, the dominant eigenvalue of the spectral radius represents the basic reproduction number $({\mathscr {R}}_{0})$ of FOM (2)

8 $$\begin{aligned} {} {\mathscr {R}}_{0} = \frac{\Lambda ^{\alpha } \beta ^{\alpha }}{(\mu _{0}^{\alpha }+\nu ^{\alpha })(\mu _{0}^{\alpha }+\mu _{1}^{\alpha }+\sigma ^{\alpha })}. \end{aligned}$$

This shows that if ${\mathscr {R}}_{0} \le 1,$ then the disease does not spread in the population and the infection dies. On the other hand, if ${\mathscr {R}}_{0} \ge 1,$ then the disease persists in the population.

The other steady state solution of FOM (2) is also known as the infective steady state (endemic equilibrium (EE)) point. Now, let ${\mathscr {E}}^{*}= ({S}^{*},{I}^{*},{R}^{*})$ represents EE point of FOM (2), where

9 $$\begin{aligned} {} S^{*} = \frac{(\mu _{0}^{\alpha }+ \mu _{1}^{\alpha }+\sigma ^{\alpha })+\Lambda ^{\alpha } \gamma ^{\alpha } }{(\beta ^{\alpha }+(\mu _{0}^{\alpha }+\nu ^{\alpha }) \gamma ^{\alpha })}~~, I^{*} = \frac{(\mu _{0}^{\alpha }+\nu ^{\alpha }) ({\mathscr {R}}_{0}-1)}{(\beta ^{\alpha }+(\mu _{0}^{\alpha }+\nu ^{\alpha }) \gamma ^{\alpha })},~\text {and}~ R^{*} =\frac{1}{\mu _{0}^{\alpha }}. (\sigma ^{\alpha } I^{*}+\nu ^{\alpha } S^{*}) \end{aligned}$$

From Eq. (9), we observe that EE point of FOM (2) depends on ${\mathscr {R}}_{0}$. That means, a positive endemic equilibrium exists if ${\mathscr {R}}_{0} > 1$.

### Local stability

#### Theorem 6

*If* ${\mathscr {R}}_{0} \le 1$, *then* ${\mathscr {E}}_{0} = \bigg (\big (\frac{\Lambda }{\mu _{0}} \big )^{\alpha },0,0 \bigg )$ *is locally asymptotically stable in* $\Omega$ for $\alpha \in (0,1]$ *and otherwise unstable.*

#### Proof

The desired Jacobian matrix at ${\mathscr {E}}_{0}$ becomes

10 $$\begin{aligned} {} J_{{\mathscr {E}}_{0}} = \left( \begin{array}{ccc} -(\mu _{0}^{\alpha }+\nu ^{\alpha }) &{}\quad \frac{^{\alpha } \beta ^{\alpha }}{\mu _{0}^{\alpha }+\nu ^{\alpha }} &{}\quad 0\\ 0 &{}\quad -(\mu _{0}^{\alpha }+\mu _{1}^{\alpha }+\sigma ^{\alpha }) &{}\quad 0\\ \nu ^{\alpha } &{}\quad \sigma ^{\alpha } &{}\quad -\mu _{0}^{\alpha }. \end{array} \right) \end{aligned}$$

The eigenvalues of Eq. (10) are

$$\begin{aligned} \lambda _{1} = -(\mu _{0}^{\alpha }+\nu ^{\alpha }), ~ \lambda _{2} = -(\mu _{0}^{\alpha }+\mu _{1}^{\alpha }+\sigma ^{\alpha }) ~\text {and} ~ \lambda _{3} = -\mu _{0}^{\alpha }. \end{aligned}$$

The three eigenvalues in the above equation have negative real parts such that the condition $|arg(\lambda _{i})| = \pi > \frac{\alpha \pi }{2}$ as defined in Definition 4 is satisfied for $i=1,2,3$, Therefore, ${\mathscr {E}}_{0}$ is locally asymptotically stable in $\Omega$ if ${\mathscr {R}}_{0} \le 1$. $\square$

#### Theorem 7

*If* ${\mathscr {R}}_{0} \ge 1$, *then* ${\mathscr {E}}^{*} = (S^{*},I^{*},R^{*})$ *is locally asymptotically stable in* $\Omega$ *for* $\alpha \in (0,1]$ *and otherwise unstable.*

#### Proof

The Jacobian matrix of the FOM (2) at EE becomes

11 $$\begin{aligned} {} J_{{\mathscr {E}}^{*}} = \left( \begin{array}{ccc} -\left( \frac{\beta ^{\alpha } I^{*}}{1+\gamma ^{\alpha } I^{*}}+(\mu _{0}^{\alpha }+\nu ^{\alpha }) \right) &{}\quad - \frac{\beta ^{\alpha } S^{*}}{(1+\gamma ^{\alpha } I^{*})^{2}} &{}\quad 0\\ \frac{\beta ^{\alpha } I^{*}}{1+\gamma ^{\alpha } I^{*}} &{}\quad \frac{\beta ^{\alpha } S^{*}}{(1+\gamma ^{\alpha } I^{*})^{2}}-{(\mu _{0}^{\alpha }+\mu _{1}^{\alpha }+\sigma ^{\alpha })} &{}\quad 0\\ \nu ^{\alpha } &{}\quad \sigma ^{\alpha } &{}\quad -\mu _{0}^{\alpha } \end{array} \right) . \end{aligned}$$

The characteristic equation of $J_{{\mathscr {E}}^{*}}$ from Eq. (11) becomes

$$\begin{aligned} \psi (t) = (\mu _{0}^{\alpha }+\lambda )(\lambda ^{2}+a_{1} \lambda +a_{2}), \end{aligned}$$

where

12 $$\begin{aligned} {} \begin{aligned}{}&a_{1} = 2 \mu _{0}^{\alpha }+\nu ^{\alpha }+\mu _{1}^{\alpha }+\sigma ^{\alpha }+\frac{\beta ^{\alpha } (\gamma ^{\alpha } I^{*2}+I^{*}-S^{*})}{(1+\gamma ^{\alpha } I^{*})^{2}}, ~\text {and}\\&a_{2} = (\mu _{0}^{\alpha }+\nu ^{\alpha })(\mu _{0}^{\alpha }+\mu _{1}^{\alpha }+\sigma ^{\alpha })+\frac{\beta ^{\alpha } ((\mu _{0}^{\alpha }+\mu _{1}^{\alpha }+\sigma ^{\alpha })(1+\gamma ^{\alpha }I^{*})I^{*}-(\mu _{0}^{\alpha }+\nu ^{\alpha })S^{*})}{(1+\gamma ^{\alpha } I^{*})^{2}}. \end{aligned} \end{aligned}$$

In Eq. (12), we see that $a_{1}>0$ if $(1+\gamma ^{\alpha } I^{*}) I^{*} > S^{*}$ and $a_{2} > 0$ if $\beta ^{\alpha } ((\mu _{0}^{\alpha }+\mu _{1}^{\alpha }+\sigma ^{\alpha })(1+\gamma ^{\alpha }I^{*})I^{*}>(\mu _{0}^{\alpha }+\nu ^{\alpha })S^{*})~ \text {when} ~ {\mathscr {R}}_{0} \ge 1$. Hence, the fractional Routh–Hurwitz condition is satisfied, therefore, the EE of FOM (2) is locally asymptotically stable in $\Omega$ for $\alpha \in (0,1]$. $\square$

### Sensitivity analysis

The sensitivity analysis of the basic reproduction number $({\mathscr {R}}_{0})$ plays a vital role in pointing out the most sensitive model parameters that take place in HBV transmission and control. Here, we study the relative contribution of each model parameter as it plays a significant role in disease control due to the fact that the disease incidence is related to ${\mathscr {R}}_{0}$. This leads us to determine the most sensitive parameters in the spread of the HBV infection. Before constructing an appropriate fractional order optimal control problem, we perform the sensitivity analysis of the fractional order system. We apply the normalized forward sensitivity index method to measure the importance of each parameter of FOM (2) in the disease incidence as defined in^32^.

We calculate the normalized sensitivity index of ${\mathscr {R}}_{0}$ with respect to the basic parameters of the model for the integer order case $(\alpha = 1)$. For example, the sensitivity index of ${\mathscr {R}}_{0}$ with respect to the parameter $\beta , ~{{\Psi }_{\beta }^{{\mathscr {R}}_{0}}}$, for the classical case $(\alpha = 1)$ is computed as follows

$$\begin{aligned} {{\Psi }_{\beta }^{{\mathscr {R}}_{0}}} = \frac{\partial }{\partial \beta } \left( \frac{\Lambda \beta }{(\mu _{0}+\nu )(\mu _{0}+\mu _{1}+\sigma )} \right) \times \left( \frac{\beta (\mu _{0}+\nu )(\mu _{0}+\mu _{1}+\sigma )}{\Lambda \beta } \right) = + 1.00 \end{aligned}$$

In the same procedure, the sensitivity indexes of ${\mathscr {R}}_{0}$ with respect to the parameters $\Lambda , \nu ~\text {and}~\sigma$ are calculated and the results obtained.

**Table 2:** Sensitivity indexes of parameters of the model for $\alpha =1$.

Parameter	Value	$\Psi _{q}^{{\mathscr {R}}_{0}}$	Parameter	Value	$\Psi _{q}^{{\mathscr {R}}_{0}}$
$\Lambda$	0.2320000	+ 1.0000	$\nu$	0.0200000	− 0.9885
$\beta$	0.0009000	+ 1.0000	$\mu _{1}$	0.0000547	− 0.0019
$\mu _{0}$	0.0002320	- 0.0134	$\sigma$	0.1200000	− 0.9976

The values of the sensitivity indexes of ${\mathscr {R}}_{0}$ with respect to basic model parameters evaluated at DFE for FOM (2) using parameter values in Table 1 are enlisted in Table 2. Based on sensitivity indices presented in Table 2, the most sensitive parameters to disease incidence are the transmission rate $(\beta )$, vaccination rate $(\nu )$, the disease-induced death rate $(\mu _{1})$, and the recovery rate $(\sigma )$ of infectious individuals. From Table 2 we observe that $\Psi _{\nu }^{{\mathscr {R}}_{0}} = - 0.9885$ which shows the parameter $\nu$ is inversely related to ${\mathscr {R}}_{0}$. This means that an increase (a decrease) of the value of $\nu$ by a small percentage will decrease (increase) ${\mathscr {R}}_{0}$ by approximately the same percentage for the fractional order $\alpha = 1$. On the other hand, the parameter $\beta$ is directly related to ${\mathscr {R}}_{0}$ which means that an increase (a decrease) of the value of $\beta$, for instance by 10%, will increase (decrease) the value of ${\mathscr {R}}_{0}$ by 10%.

However, the sensitivity index of FOM (2) parameters is determined by the derivative order $\alpha \in (0,1)$. The effect of the fractional derivative order $\alpha \in (0,1)$ on model parameters $\nu ~\text {and}~ \sigma$ has a considerable impact on the sensitivity index of model parameters. The sensitivity indexes of $\nu$ and $\sigma$ exhibit their impact on ${\mathscr {R}}_{0}$ with respect to $\alpha$, being very sensitive to the variation of $\alpha$. The sensitivity indexes $\mu _0$ and $\mu _1$, on the other hand, are substantially less sensitive to variations in the fractional-order $\alpha$. The fractional order model is less sensitive than the integer order model because the model’s sensitivity reduces in absolute value as the fractional derivative order $\alpha$ decreases.

In fact, the parameters whose index value for $\alpha =1$ is close to zero, do not vary much if we consider lower values of $\alpha$. On the other hand, parameters whose index value in Table 2 is not as close to zero as the previous one vary significantly if we consider lower values of $\alpha$. As a result, for the better elimination of HBV infection, it is important to increase the recovery rate $(\sigma )$ and vaccination rate $(\nu )$. After considering the sensitivity analyses, it is determined that reasonable actions will be taken to control the transmission of the disease.

## Fractional optimal control problem

In this section, we develop a fractional optimal control problem (FOCP) to prevent HBV infection. We apply the fractional optimal control methods to formulate a control mechanism for the spread of HBV infection based on sensitivity analysis. From the sensitivity analysis, it is obtained that the vaccination rate $\nu$ and the recovery rate $\sigma$ have a high magnitude of sensitivity indexes. Hence our main purposes are to reduce HBV infection using vaccination $(\nu =u_{1})$ of susceptible individuals, maximize the recovered individuals using treatment $(u_{2})$ of infectious individuals, and simultaneously, reduce the associated cost of the control measures. The optimality control concepts are utilized to formulate the control system. Our main objective here is to minimize the objective function *J* by finding a pair of optimal controls $u_{1}^{*} ~\text {and}~ u_{2}^{*}$ such that

13 $$\begin{aligned} {} \begin{aligned}{}&J(u_{1}^{*}, u_{2}^{*}) = \min {J(u_{1}, u_{2})},\\&J(u_{1},u_{2}) = \int _{0}^{T}{\bigg (A I(t)+\frac{1}{2}B_{1}u_{1}^{2}(t)+\frac{1}{2}B_{2}u_{2}^{2}(t) \bigg )dt}, \end{aligned} \end{aligned}$$

subject to the fractional order system

14 $$\begin{aligned} {} {\left\{ \begin{array}{ll} {_{0}^{C}}{D_{t}^{\alpha }}{S(t)} = \Lambda ^{\alpha }-\frac{\beta ^{\alpha } S(t) I(t)}{1+\gamma ^{\alpha } I(t)}-(\mu _{0}^{\alpha }+u_{1}(t))S(t),\\ {_{0}^{C}}{D_{t}^{\alpha }}{I(t)} = \frac{\beta ^{\alpha } S(t) I(t)}{1+\gamma ^{\alpha } I(t)}-(\mu _{0}^{\alpha }+\mu _{1}^{\alpha } +\sigma ^{\alpha }+u_{2}(t))I(t),\\ {_{0}^{C}}{D_{t}^{\alpha }}{R(t)} = (u_{2}(t)+\sigma ^{\alpha }) I(t)+u_{1}(t) S(t)-\mu _{0}^{\alpha } R(t) \end{array}\right. } \end{aligned}$$

along the following non-negative initial conditions (ICs)

15 $$\begin{aligned} {} S(0) \ge 0, I(0) \ge 0, R(0) \ge 0 \end{aligned}$$

In Eq. (13), the positive constant measures the weight constant for the infectious class *I* whereas $B_{1} ~\text {and} ~B_{2}$ are a measure of the relative cost of the interventions associated with the vaccination and treatment controls, respectively. Here, the values $\frac{1}{2}B_{1}u_{1}^{2} ~\text {and}~\frac{1}{2}B_{2}u_{2}^{2}$ describe the costs associated with the vaccination and treatment intervention strategies, respectively. It is supposed that the costs are proportional to the square of the corresponding control functions. We also assumed that the set of admissible control functions

16 $$\begin{aligned} {} {\mathscr {O}}= \{(u_{1}(t), u_{2}(t)) :0 \le u_{i} \le u_{i}^{\max }\}, \end{aligned}$$

where $u_{i}$ is Lebesgue measurable over [0, *T*], $u_{i}^{\max }$ is the maximum value of control measures and *T* is a fixed final time.

The optimal control must satisfy the necessary conditions that are formulated by Pontryagin’s Maximum Principle^33^. This principle converts the FOCP (13)–(15) into a problem of minimizing point-wise a Hamiltonian with respect to the control $u(t)=(u_{1}(t),u_{2}(t))$ as

$$\begin{aligned}{}&{\mathscr {H}}(t,z(t),u(t),\lambda (t)) = \lambda ^{T}(t) f(t,z(t),u(t))+L(t,z(t),u(t)), \end{aligned}$$

where $f(t,z(t),u(t)) = {_{0}^{C}}{D_{t}^{\alpha }}{z(t)}, ~z(t)=(S(t),I(t),R(t))~\text {and}~ L(t,z(t),u(t))=A_{1}I(t)+\frac{1}{2}B_{1}u_{1}^{2}+\frac{1}{2}B_{2}u_{2}^{2} ~~\text {is the Lagrangian,}~ \lambda (t) = (\lambda _{1}(t), \lambda _{2}(t), \lambda _{3}(t))$ is the adjoint variable of the fractional order system.

### Theorem 8

*Let* $S^{*},I^{*}$ *and* $R^{*}$ *be the optimal state solutions associated with the optimal controls* $u_{1},u_{2}$ *which* $min{J(u_{1},u_{2})}$ *in the FOCP* (13)–(15). *Then, there exists a non-trivial absolutely continuous mapping* $\lambda :[0,T] \rightarrow {\mathbb {R}}_{+}, \lambda =(\lambda _{1},\lambda _{2},\lambda _{3})$, *such that*

17 $$\begin{aligned} {} \begin{aligned}{}&{_{0}^{C}}{D_{t}^{\alpha }}{\lambda _{1}(t^{'})}=(\lambda _{1}-\lambda _{2}) \left( \frac{\beta ^{\alpha } I}{1+\gamma ^{\alpha } I} \right) +(\lambda _{1}-\lambda _{3})u_{1}+\lambda _{1} \mu _{0}^{\alpha },\\&{_{0}^{C}}{D_{t}^{\alpha }}{\lambda _{2}(t^{'})} = (\lambda _{1}-\lambda _{2}) \left( \frac{\beta ^{\alpha } S}{(1+\gamma ^{\alpha } I)^{2}} \right) +(\lambda _{2}-\lambda _{3})(\sigma ^{\alpha }+u_{2})+\lambda _{2}(\mu _{0}^{\alpha }+\mu _{1}^{\alpha })-A,\\&{_{0}^{C}}{D_{t}^{\alpha }}{\lambda _{3}(t^{'})} = \lambda _{3} \mu _{0}^{\alpha }, \end{aligned} \end{aligned}$$

*where* $t^{'}=T-t$ *with transversality conditions*

18 $$\begin{aligned} {} \lambda _{1}(T) = \lambda _{2}(T) =\lambda _{3}(T) = 0. \end{aligned}$$

*Furthermore, the optimal solution* $(u_{1}^{*},u_{2}^{*})$ *that minimizes the fractional optimal control in* ${\mathscr {O}}$ *such that*

19 $$\begin{aligned} {} \begin{aligned}{}&u_{1}^{*}(t) = \min \bigg \{ \max \bigg \{ 0, \frac{(\lambda _{3}-\lambda _{2}) S^{*}}{B_{1}} \bigg \}, 1 \bigg \},~\text {and} \\&u_{2}^{*}(t) = \min \bigg \{ \max \bigg \{ 0, \frac{(\lambda _{1}-\lambda _{3}) I^{*}}{B_{2}} \bigg \}, 1 \bigg \}. \end{aligned} \end{aligned}$$

### Proof

In the fractional optimal control, the state and the control variables are positive. Thus, the necessary convexity of the objective functional in the control pair is satisfied. The admissible set ${\mathscr {O}}$ is compact. Thus, the Hamiltonian $H(t,z,u,\lambda )$ can be written as

20 $$\begin{aligned} {} \begin{aligned} {\mathscr {H}}(t,z,u,\lambda )&= A I(t) + \frac{1}{2} B_{1} u_{1}^{2}+\frac{1}{2} B_{2} u_{2}^{2} \\&\quad +\lambda _{1} \left( \Lambda ^{\alpha }-\frac{\beta ^{\alpha } S I}{1+\gamma ^{\alpha } I}-(\mu _{0}^{\alpha }+u_{1}) S \right) \\&\quad +\lambda _{2} \left( \frac{\beta ^{\alpha } S I}{1+\gamma ^{\alpha } I}-(\mu _{0}^{\alpha }+\mu _{1}^{\alpha }+\sigma ^{\alpha }+u_{2}) I \right) \\&\quad +\lambda _{3} \left( u_{1} S+(\sigma ^{\alpha }+u_{2}) I-\mu _{0}^{\alpha } R \right) \end{aligned} \end{aligned}$$

Using Theorem 2 the right Riemann–Liouville fractional derivative of Eq. (20) over [0, *T*] becomes

21 $$\begin{aligned} {} \begin{aligned} {_{t}^{RL}}{D_{T}^{\alpha }}{\lambda _{1}(t)}&= -\frac{\partial {\mathscr {H}}(t,z^{*}(t),\lambda ^{*}(t),u^{*}(t))}{\partial S^{*}}\\&= (\lambda _{1}-\lambda _{2}) \left( \frac{\beta ^{\alpha } I}{1+\gamma ^{\alpha } I} \right) +(\lambda _{1}-\lambda _{3})u_{1}+\lambda _{1} \mu _{0}^{\alpha },\\ {_{t}^{RL}}{D_{T}^{\alpha }}{\lambda _{2}(t)}&= -\frac{\partial {\mathscr {H}}(t,z^{*}(t),\lambda ^{*}(t),u^{*}(t))}{\partial I^{*}}\\&= (\lambda _{1}-\lambda _{2}) \left( \frac{\beta ^{\alpha } S}{(1+\gamma ^{\alpha } I)^{2}}\right) +(\lambda _{2}-\lambda _{3})(\sigma ^{\alpha }+u_{2})+\lambda _{2}(\mu _{0}^{\alpha }+\mu _{1}^{\alpha })-A, ~\text {and}\\ {_{t}^{RL}}{D_{T}^{\alpha }}{\lambda _{3}(t)}&= -\frac{\partial {\mathscr {H}}(t,z^{*}(t),\lambda ^{*}(t),u^{*}(t))}{\partial R^{*}} = \lambda _{3} \mu _{0}^{\alpha }, \end{aligned} \end{aligned}$$

where $0 \le t \le T$. Equivalently, Eq. (21) can be written in the right Caputo-fractional derivative using Theorem 2, which yields

22 $$\begin{aligned} {} \begin{aligned}{}&{_{t}^{C}}{D_{T}^{\alpha }}{\lambda _{1}(t)} = (\lambda _{1}-\lambda _{2}) \left( \frac{\beta ^{\alpha } I}{1+\gamma ^{\alpha } I} \right) +(\lambda _{1}-\lambda _{3})u_{1}+\lambda _{1} \mu _{0}^{\alpha },\\&{_{t}^{C}}{D_{T}^{\alpha }}{\lambda _{2}(t)} = (\lambda _{1}-\lambda _{2}) \left( \frac{\beta ^{\alpha } S}{(1+\gamma ^{\alpha } I)^{2}} \right) +(\lambda _{2}-\lambda _{3})(\sigma ^{\alpha }+u_{2})+\lambda _{2}(\mu _{0}^{\alpha }+\mu _{1}^{\alpha })-A,\\&{_{t}^{C}}{D_{T}^{\alpha }}{\lambda _{3}(t)} = \lambda _{3} \mu _{0}^{\alpha } \end{aligned} \end{aligned}$$

According to Lemma 1, Eq. (22) can be converted into the left Caputo fractional derivative as follow:

$$\begin{aligned} \begin{aligned} {_{0}^{C}}{D_{t}^{\alpha }}{\lambda _{1}(t')}&= (\lambda _{1}(t')-\lambda _{2}(t')) \left( \frac{\beta ^{\alpha } I(t')}{1+\gamma ^{\alpha } I (t')} \right) +(\lambda _{1}(t')-\lambda _{3}(t'))u_{1}(t')+\lambda _{1}(t') \mu _{0}^{\alpha },\\ {_{0}^{C}}{D_{t}^{\alpha }}{\lambda _{2}(t')}&= (\lambda _{1}(t')-\lambda _{2}(t')) \left( \frac{\beta ^{\alpha } S(t')}{(1+\gamma ^{\alpha } I(t'))^{2}} \right) +(\lambda _{2}(t')-\lambda _{3}(t'))(\sigma ^{\alpha }+u_{2}(t'))\\&\quad +\lambda _{2}(t')( \mu _{0}^{\alpha }+\mu _{1}^{\alpha })-A,\\ {_{0}^{C}}{D_{t}^{\alpha }}{\lambda _{3}(t')}&= \lambda _{3}(t') \mu _{0}^{\alpha }, ~~~\text {where}~ t'=T-t \end{aligned} \end{aligned}$$

For the optimal control variables $u_{1}^{*} ~\text {and}~ u_{2}^{*}$, we partially differentiate the Hamiltonian ${\mathscr {H}}$ using Eq. (21) with respect to $u_{i}^{*}~(i=1,2)$. To this end, we solve the system $\frac{\partial {\mathscr {H}}}{\partial u_{i}^{*}} = 0$ to obtain the necessary optimality condition for the finite-dimensional optimization problem, when the control pairs $u_{1} ~\text {and}~ u_{2}$ are in the interior of ${\mathscr {O}}$ as follows.

23 $$\begin{aligned} {} \begin{aligned}{}&\frac{\partial {\mathscr {H}}(t,z^{*}(t),\lambda ^{*}(t),u^{*}(t))}{\partial u_{1}^{*}} = B_{1}u_{1}^{*}+\lambda _{1}S^{*}-\lambda _{3} S^{*} = 0,~\text {and} \\&\frac{\partial {\mathscr {H}}(t,z^{*}(t),\lambda ^{*}(t),u^{*}(t))}{\partial u_{2}^{*}} - B_{1}u_{2}^{*}+\lambda _{2} I^{*}+\lambda _{3} I^{*} = 0. \end{aligned} \end{aligned}$$

Upon simplification of Eq. (23), we obtain

24 $$\begin{aligned} {} u_{1}^{*} = \frac{(\lambda _{1}-\lambda _{3}) S^{*}}{B_{1}}, ~~~~\text {and}~~~~~u_{2}^{*} = \frac{(\lambda _{2}-\lambda _{3}) I^{*}}{B_{2}}. \end{aligned}$$

Now, taking the lower and upper bounds of Eq. (24) we obtain

$$\begin{aligned} \overline{u_{1}} = {\left\{ \begin{array}{ll} 0, &{} \text {if} ~u_{1}^{*}~ \le 0,\\ u_{1}^{*}, &{} \text {if} ~ 0< u_{1}^{*}<1,\\ 1, &{} \text {if} ~ u_{1}^{*} \ge 0, \end{array}\right. } ~~~~\text {and}~~~~ \overline{u_{2}} = {\left\{ \begin{array}{ll} 0, &{} \text {if} ~ u_{2}^{*} \le 0,\\ u_{1}^{*}, &{} \text {if} ~ 0< u_{1}^{*}<1,\\ 1, &{} \text {if} ~ u_{1}^{*} \ge 0. \end{array}\right. } \end{aligned}$$

Hence, the maximality optimal controls ${\overline{u}}_{1} ~\text {and}~ {\overline{u}}_{2}$ situations become

$$\begin{aligned} \begin{aligned}{}&\overline{u_{1}} = \max \big \{0, \min \big \{1,u_{1}^{*} \big \} \big \}, \\&\overline{u_{2}} = \max \big \{0, \min \big \{1,u_{2}^{*} \big \} \big \}. \end{aligned} \end{aligned}$$

This achieves the maximality condition

$$\begin{aligned} {\mathscr {H}}(t,z^{*}(t),\lambda (t), u^{*}(t)) = \min _{0 \le u \le 1}{{\mathscr {H}}(t,z^{*}(t),\lambda (t), u(t))}. \end{aligned}$$

$$\square$$

## Numerical results

In this section, we utilize Pontryagin’s Maximum Principle to numerically solve the FOCP (15). The fractional optimal control is obtained by solving the optimality system. We use MATLAB software with an iterative scheme used for solving the optimality system. We start by solving the state equations with a guess for the controls over the simulated time using forward fractional Euler’s method. Because of the transversality conditions, the co-state equations are solved by the backward fractional Euler’s method using the current iteration solutions of the state equations. Then the controls are updated by using a convex combination of the previous controls. This process is repeated and iterations stop if the values of the unknowns at the previous iterations are almost coincident with the ones of the previous iteration, that is until convergence is achieved. The solutions to the fractional control problem were performed and successfully confirmed by a classical forward-backward sweep method when $\alpha = 1$. Before performing the numerical experiments of the proposed FOM (2) and its extension to FOCP (13)–(15), we will present the numerical scheme to carry out the numerical simulation.

Consider the interval of the solutions to fractional optimal control to be [0, *T*], which is partitioned into *N* equispaced grids, each of which has length $h = \frac{T}{N}$. Thus, the nodes become $t_{n} = nh, (n=1,2,\dots ,N)$. Under the numerical scheme, we present the numerical solution of the nonlinear fractional differential equations (FDEs) by approximating solutions. Consider the subsequent initial value nonlinear (FDE) problem

$$\begin{aligned}{}&{_{t_{0}}^{C}D_{t}^{\alpha }}{z(t)} = f(t,z(t)), ~~ 0 \le t \le T\\&z^{(k)}(0) = z_{0}^{k}, ~~ k=0,1,2, \dots , [\alpha ]-1. \end{aligned}$$

For the above equation, the numerical scheme is formulated as

$$\begin{aligned} z_{k} = z_{0}+\frac{h^{\alpha }}{\Gamma (\alpha +1)} {\sum _{j=0}^{k}{(b_{j,k})f(t_{j},z(t_{j}))}}, \end{aligned}$$

where $$b_{j,k} = (k-j)^{\alpha }-(k-j-1)^{\alpha }$$.

The numerical solution of the Caputo fractional derivative of Eq. (14) becomes

25 $$\begin{aligned} {} \begin{aligned}{}&S(t_{k}) = S(0)+\frac{h^{\alpha }}{\Gamma (\alpha +1)} \sum _{j=0}^{k-1}{b_{j,k}} \left( \Lambda ^{\alpha }-\frac{\beta ^{\alpha } S(t_{j}) I(t_{j})}{1+\gamma ^{\alpha } I(t_{j})}-(\mu _{0}^{\alpha }+u_{1}(t_{j}))S(t_{j}) \right) ,\\&I(t_{k}) = I(0)+\frac{h^{\alpha }}{\Gamma (\alpha +1)} \sum _{j=0}^{k-1}{b_{j,k}} \left( \frac{\beta ^{\alpha } S(t_{j}) I(t_{j})}{1+\gamma ^{\alpha } I(t_{j})}-(\mu _{0}^{\alpha }+\mu _{1}^{\alpha }+\sigma ^{\alpha }+u_{2}(t_{j}))I(t_{j}) \right) ,\\&R(t_{k}) = R(0)+\frac{h^{\alpha }}{\Gamma (\alpha +1)} \sum _{j=0}^{k-1}{b_{j,k}} \left( (\sigma ^{\alpha }+u_{2}(t_{j}))I(t_{j})+u_{1}(t_{j}) S(t_{j})-\mu _{0}^{\alpha } R(t_{j}) \right) . \end{aligned} \end{aligned}$$

In the same procedure, the solutions to the adjoint equations become

26 $$\begin{aligned} {} \begin{aligned}{}&\lambda _{1}(t_{k-1}^{'}) = \frac{h^{\alpha }}{\Gamma (\alpha +1)} \sum _{j=0}^{n}{b_{j,k+1}} \bigg [(\lambda _{1}-\lambda _{2}) \left( \frac{\beta ^{\alpha } }{1+\gamma ^{\alpha } I} \right) +(\lambda _{1}-\lambda _{3}) u_{1}+\lambda _{1} \mu _{0}^{\alpha } \bigg ], \\&\lambda _{2}(t_{k-1}^{'}) = \frac{h^{\alpha }}{\Gamma (\alpha +1)} \sum _{k=0}^{j}{b_{j,k+1}} \bigg [(\lambda _{1}-\lambda _{2}) \left( \frac{\beta ^{\alpha } }{(1+\gamma ^{\alpha } I)^{2}} \right) +(\lambda _{2}-\lambda _{3})(\sigma ^{\alpha }+u_{2})+\lambda _{2}(\mu _{0}^{\alpha }+\mu _{1}^{\alpha })-A \bigg ], \\&\lambda _{3}(t_{k-1}^{'}) = \frac{h^{\alpha }}{\Gamma (\alpha +1)} \sum _{j=0}^{k}{b_{j,k+1}} \left( \lambda _{3}(t_{j}) \mu _{0}^{\alpha } \right) , \end{aligned} \end{aligned}$$

where $$b_{j,k+1} = (k-j)^{\alpha }-(k-j+1)^{\alpha }$$.

## Discussion

**Figure 1:**
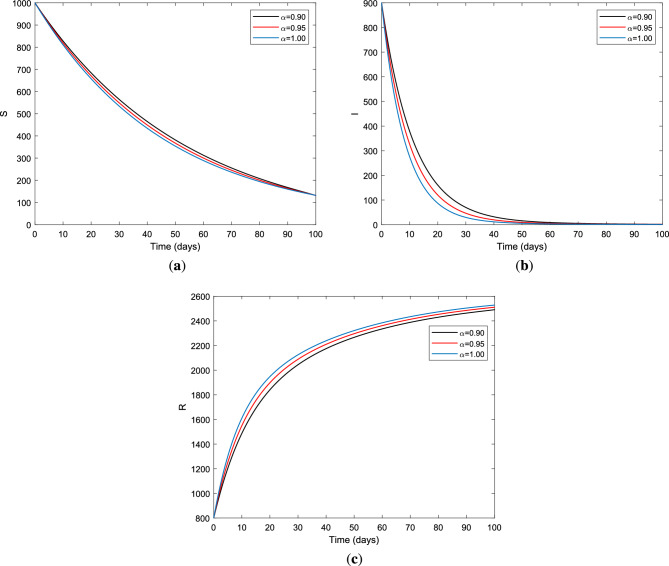
Evolution of (**a**) susceptible, (**b**) infected, and (**c**) recovered trajectories for FOM (2) with different fractional derivative order $\alpha$ for ${\mathscr {R}}_{0}=0.0858>1$ using parameter values in Table 1.

**Figure 2:**
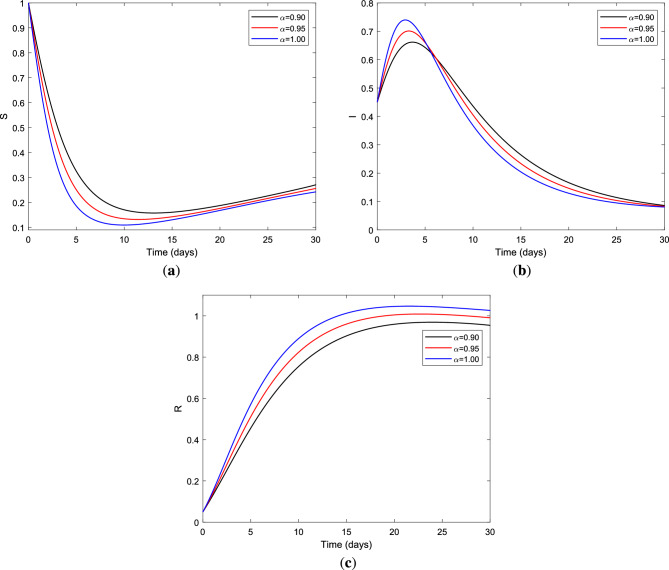
Dynamic behavior of (**a**) susceptible class, (**b**) infected class and (**c**) recovered class for FOM (2) with different fractional order $\alpha$ for ${\mathscr {R}}_{0} = 2.8846 > 1$ using parameter value set *S*.

**Figure 3:**
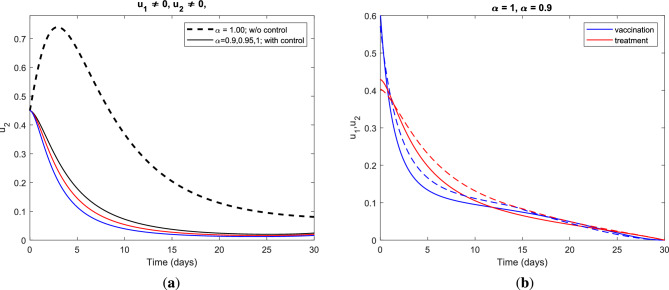
(**a**) Impact of the optimal combination of vaccination and treatment control measures on infectious class and (**b**) the control profile for vaccination $u_{1}$ and treatment $u_{2}$ strategy using parameter value set *S* with different fractional derivative order $\alpha$.

**Figure 4:**
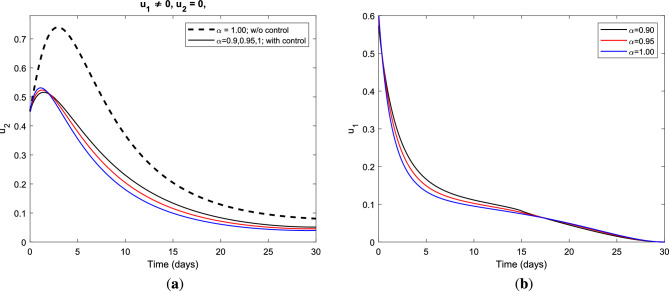
(**a**) Impact of the optimal use of vaccination only control strategy on infectious class and (**b**) the control profile for vaccination $u_{1}$ strategy using parameter value set *S* with different fractional derivative order $\alpha$.

**Figure 5:**
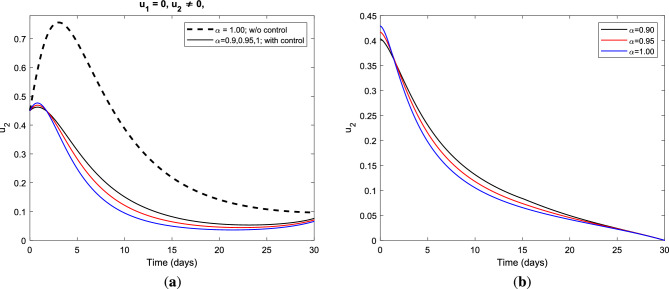
(**a**) Impact of the optimal use of treatment only strategy on infectious class and (**b**) the control profile for treatment $u_{2}$ strategy using parameter value set *S* with different fractional derivative order.

In this section, we present plots for solutions of FOM (2) for step size $h = 0.01$ with different fractional order $\alpha = 0.90,~\alpha = 0.95,~\text {and}~\alpha = 1.00$, considering the time range is [0, 30] for simulation purposes with initial population size to be (1, 0.45, 0.05). These plots help us to verify the stability of the disease-free equilibrium and endemic equilibrium of the model. Figures 1, 2, 3, 4 and 5 demonstrate the behavior of solution curves of FOM (2) and fractional optimal control problem for different fractional order derivatives with two control measures. We first consider for the case ${\mathscr {R}}_{0} = 0.0858<1$ using the parameter values in Table 1. This implies that the disease will wipe out the population in the passage of time even if no intervention is applied to the system^15^. It is seen in Fig. 1a that only the susceptible population survives and in Fig. 1b the infectious individuals are going extinct. This certifies that disease-free equilibrium is locally asymptotically stable for ${\mathscr {R}}_{0}<1$ which in turn verifies the analytical results presented in Theorem 6. It is obvious that as the fractional order $\alpha$ decreases in the long run, there are more susceptible individuals in the case of fractional order derivatives than in the case of integer order derivatives as depicted in Figs. 1 and 2.

Next, we simulated the endemic trajectory of fractional optimal control for different fractional order values using parameter value set $S=\{\Lambda , \beta , \nu , \mu _{0}, \sigma , \mu _{1}, \gamma \}$ whose values are $\Lambda =0.03, \beta = 0.75, \mu _{0}=0.02, \sigma = 0.15, \nu =0.02, \mu _{1}=0.025 ~\text {and}~\gamma =0.5$ in the absence of treatment and vaccination control measures, which yield ${\mathscr {R}}_{0} = 2.8846 > 1$. This scenario implies that the disease is endemic regardless of the fractional derivative $\alpha$, which indicates the disease will persist if not properly managed. Indeed, a lack of understanding regarding HBV transmission dynamics causes a delay in identifying prior phases of the disease. As a result, after knowing about HBV transmission modes, susceptible persons should take precautions or get vaccinated against HBV. This is related to the memory index of the system. Generally, lower values of the memory index correspond to increased system knowledge. Based on the memory indexes, the evolution of $S(t),~I(t)~\text {and}~R(t)$ of the fractional order HBV model are plotted. In Fig. 2, it is seen that the effects of fractional orders are distinctive; the solution curves for $\alpha \in (0,1)$ show slowly in the epidemic peak and flatten faster. In particular, Fig. 2b shows the number of infectious individuals increases when the order of differentiation increases in the absence of control measures. These results reveal that if necessary interventions are not taken at a certain time, the HBV disease continues to persist in the population. So, robust strategies are required to control the transmission dynamics of the disease.

It is known that a reduction in the infectious class has a direct impact on the reduction of the HBV infection in the populations. Moreover, the infectious populations are the focus of this study. This is because infectious individuals are the population at risk of being controlled before the disease moves to the next stage of infection and progresses to cirrhosis. For this matter, we applied two control measures for the duration of one month using vaccination of susceptible individuals and treatment of infected individuals.

Figure 3 demonstrates the impact of vaccination and treatment applied to the HBV infectious class and its contour profile plot for a period of at least one month with different values of the fractional order $\alpha$.

In Fig. 3a, it is seen that the number of infectious individuals decreases considerably under this optimal policy for all considered values of index memory $\alpha$. The combination of vaccination and treatment control measures gives the best alternative for preventing HBV infection over the specified duration of the intervention period and reduces the progression of cirrhosis effectively. Figure 3b illustrates the control profile for vaccination and treatment strategy with optimal value in the range $(0,0.6) ~\text {and}~(0,0.5)$, respectively, for fractional derivative $\alpha = 1.00~\text {(solid) and}~\alpha = 0.90 ~\text {(dashed)}$. On the other hand, the graphics in Fig. 4 show the impact of the vaccination-only strategy applied to the fractional optimal control system and the control profile for vaccination control measure for different fractional order $\alpha$. Moreover, Fig. 4a illustrates the impact of vaccination-only strategy on the dynamic behavior of HBV infection which gives a better alternative for preventing HBV and reducing the next stage of the disease. As clearly seen from the graphics, vaccination alone cannot completely control the spread of the disease but it significantly reduces the burden of HBV infection in the population which in turn reduces the progression of cirrhosis.

Figure 4b represents the control profile for the vaccination-only strategy applied to fractional optimal control in the absence of treatment control measure with variation in the order of differentiation. We observe that the vaccination control strategy is affected by the memory effect of the system. As we decrease the index of memory from $\alpha = 1.00 ~\text {to}~ \alpha = 0.90$ in the long run the administration of the vaccination control measure increases for about 15 days and then decreases afterward. In order to explore the dynamic behavior of HBV disease under the treatment control measure, we simulated the graphics for fractional optimal control in the absence of vaccination control measure as seen in Fig. 5. Figure 5a illustrates the effect of the treatment-only strategy on the infectious class which shows a positive impact in the prevention of HBV infection. Under this optimal policy, the number of infectious individuals gradually decreases as the order of fractional derivatives increases. This strategy alone cannot sufficiently minimize the burden of HBV infection, however, it gives a better alternative and most cost-effective in reduce the progression of the disease into the next stage. It is seen that Fig. 5b demonstrates the control profile of the treatment-only strategy in the absence of a vaccination control measure.

In many infectious diseases, fractional derivative offers deeper insight into the system and captures the distinct memory effects of the system. In general, a lower value of fractional order derivative corresponds to less infectious individuals over the period of intervention. The number of infectious individuals is smaller in the control case for various fractional derivative orders than in the case without control. This is because the desired goal of the control measures is to reduce the number of infectious individuals and minimize the associated cost of intervention. In conclusion, the graphics clearly illustrate the effect as well as the desired goal of the controls. There is obviously a significant difference between the controlled and without-control cases. This positive influence suggests that over the intervention period, the control technique is useful in controlling the disease.

## Cost-effectiveness analysis

Cost-Effectiveness Analysis (CEA) is an economic analysis of cost that helps us to compare the relative cost of two or more alternative interventions to determine and propose the most cost-effective strategy to implement with limited resources. It is important to compare the results of different control measures, with the help of calculating the Incremental Cost Effectiveness Ratio (ICER)^34^. To evaluate the cost and effectiveness of fractional optimal control for the entire intervention period, we compute the total cases averted, the total cost associated with intervention strategies, and the average cost-effectiveness ratio. To do this, we analyze the cost-effectiveness of all alternative combinations of $u_{1} ~\text {and}~u_{2}$ which is achieved: by *Strategy* **a** by implementing both controls $(u_{1}, u_{2} \ne 0)$, *Strategy* **b** by implementing only vaccination $(u_{1} \ne 0 ~\text {and}~ u_{2} = 0)$, and *Strategy* **c** by implementing only treatment $(u_{1}=0 ~\text {and}~ u_{2} \ne 0)$. This ratio is used to compare the differences between the costs and health benefits of two alternative intervention strategies that compete for the same resources and is defined as

27 $$\begin{aligned} {} ICER = \frac{\text {Difference between cost benefits}}{\text {Difference between health benefits}} \end{aligned}$$

Following^35^ the function $F :{\mathbb {R}}^{+} \rightarrow [0,1]$ known as the efficacy function is used to measure the proportional differences in the number of infected individuals after the application of the treatment and vaccination compartment by comparing the number of infectious individuals at *t* with its initial value *I*(0) is defined as

28 $$\begin{aligned} {} F(t) = \frac{I(0)-I^{*}(t)}{I(0)} = 1-\frac{I^{*}(t)}{I(0)}. \end{aligned}$$

**Figure 6:**
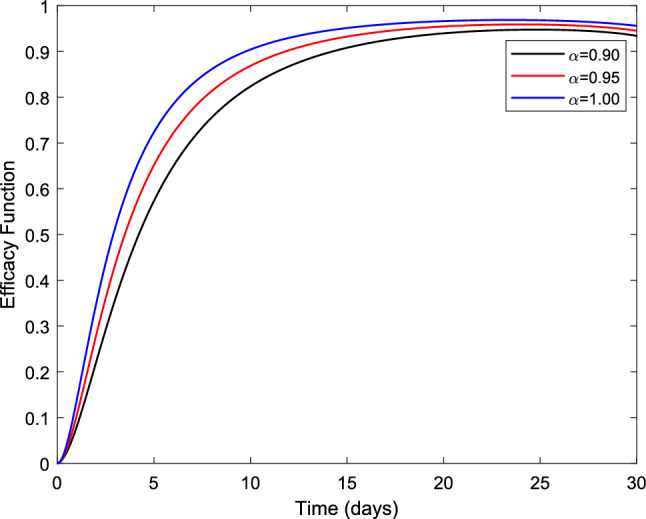
Evolution of the efficacy function of the fractional optimal control problem with different values of the fractional order $\alpha$.

In Eq. (28), the curve $I^{*}(t)$ is the optimal solution associated with the fractional optimal control and *I*(0) is the initial condition. We observe from Fig. 6 that the efficacy function exhibits the inverse tendency of infected individuals and is the highest when *F*(*t*) is unity.

The total cases averted (*AV*) by the intervention during the time period *T* is given by

29 $$\begin{aligned} {} AV = \int _{0}^{T}{I^{*}(t)dt}. \end{aligned}$$

In Eq. (29), the trajectory $I^{*}(t)$ is the optimal solution associated with the fractional optimal control. The quantity *I*(0) represents the corresponding initial condition where this initial condition is obtained as the equilibrium proportion ${\tilde{I}}(t)$ of FOM (2) with no post-exposure intervention does not depend on time. Thus, $T \times I(0) = \int _{0}^{T}{{\tilde{I}}(t)dt}$ represents the total infectious cases over a period of *T* days.

Effectiveness is the proportion of cases averted on the total cases possible under no intervention and given as^32,35^.

30 $$\begin{aligned} {} {\overline{F}} = \frac{AV}{T \times I(0)} = 1 - \frac{\int _{0}^{T}{I^{*}(t)dt}}{T \times I(0)}. \end{aligned}$$

Eq. (30) is used to compare different epidemiological scenarios, in which we choose dimensionless measures for effectiveness.

The total cost (*TC*) associated with the intervention is given by

31 $$\begin{aligned} {} TC = \int _{0}^{T}{(C_{2} u_{1} S(t)+C_{3} u_{2} I(t))dt}. \end{aligned}$$

In Eq. (31), the constants $C_{1}~\text {and}~C_{2}$ correspond to the per person unit cost of vaccination of susceptible population and treatment of infected population, respectively.

Following^35^, we define the average cost-effectiveness ratio (*ACER*) as

32 $$\begin{aligned} {} ACER = \frac{TC}{AV}. \end{aligned}$$

Eq. (32) deals with a single intervention strategy and evaluates that intervention against no intervention or current practices.

Tables 3, 4 and 5 summarize the cost-effectiveness measures of fractional optimal control with different combinations of controls.

**Table 3:** Summary of cost-effectiveness measures of hepatitis B disease with optimal use of vaccination and treatment control measures.

$\alpha$	AV	TC	ACER	${\overline{F}}$
1.00	696.1342	431.6051	0.6201	0.7162
0.95	707.4610	377.2551	0.5333	0.7509
0.90	708.8724	328.3996	0.4633	0.7814

Based on the model simulation results and using Tables 3, 4 and 5, we rank the strategies in order of increasing effectiveness for the classical order $\alpha = 1$.

**Table 4:** Incremental cost-effectiveness ratio of hepatitis B disease in the case of classical order $\alpha =1.00$.

Strategy	AV	TC	ACER	ICER
**b**	375.6922	245.6078	0.6540	0.6540
**c**	573.1921	318.5051	0.5557	0.3691
**a**	696.1342	431.6051	0.6201	0.9199

$$\begin{aligned}{}&\text {ICER}({\textbf {b}}) = \text {ACER}({\textbf {b}}) = 0.6540 \\&\text {ICER}({\textbf {c}}) = \frac{TC({\textbf {c}})-TC({\textbf {b}})}{AV({\textbf {c}})-AV({\textbf {b}})} = \frac{72.8973}{197.4999} = 0.3691 \\&\text {ICER}({\textbf {a}}) = \frac{TC({\textbf {c}})-TC({\textbf {a}})}{AV({\textbf {a}})-AV({\textbf {b}})} = - \frac{113.1}{122.9421} = 0.9199 \\ \end{aligned}$$

The comparison between strategies **b** and **c** shows that ICER(**c**) < ICER(**b**). The lower ICER value corresponds to strategy **b** strongly dominating strategy **c**, which is more costly and less effective. Therefore, strategy **b** is excluded from the set of alternative interventions so that it does not consume limited resources. We arrange the remaining strategies by increasing effectiveness and recalculating the ICER for strategies **a** and **c**. Again, the comparison between strategies **a** and **c** show that ICER(**c**) < ICER(**a**). This implies that strategy **a** strongly dominated strategy **c**. It is more costly and consumes limited resources, therefore, we exclude strategy **a** from the list of intervention strategies. With this result, we conclude that strategy **c** (treatment of infectious individuals) has the least ICER. Therefore, strategy **c** is more cost-effective than strategy **a** and **b** in the case of the classical order model as indicated in Table 4.

The ICER was calculated in the same way for the fractional order derivative situations. Table 5 compares the cost-effectiveness of strategy ${\textbf {c}}$ for fractional order values $\alpha = 0.95 ~\text {and}~\alpha = 0.90$, yielding ICER values. As the effectiveness ratio drops as the derivative order decreases, we exclude the intervention with the highest total cost first. As a result, $\alpha = 1.00~\text {and}~\alpha = 0.95$ are excluded. Finally, strategy ${\textbf {c}}~\text {(treatment of infectious individuals)}$ with fractional derivative order $\alpha = 0.90$ is the most cost-effective intervention. This conclusion, however, should be interpreted with caution due to ambiguities surrounding the parameter values, but it provides crucial deeper insight into the prevention of HBV disease with variation in the order of differentiation.

**Table 5:** Incremental cost-effectiveness ratio for strategy ${\textbf {c}}$ with variation in the order of differentiation.

$\alpha$	AV	TC	ACER	ICER
1.00	573.1921	318.5051	0.5557	0.3691
0.95	580.0607	285.4186	0.4920	0.3759
0.90	581.6606	253.4736	0.4358	0.3649

## Conclusion

In this paper, we derived and analyzed a deterministic fractional order model for the transmission of hepatitis B disease that includes treatment and vaccination control measures with a saturated incidence rate. It is known that treatment and vaccination of hepatitis B disease reduce the risk of progression and so it is desirable to apply control measures in these efforts to prevent the disease. The proposed model consists of susceptible (S), infected (I), and recovered (R) individuals, which are considered to be the most basic components of HBV infection.

The positivity of solutions and invariant region of the fractional order model are discussed to show the biological significance of the system. The equilibrium points for disease-free steady state and infected steady state are computed, and an investigation of their local stability is performed. Thus, the condition under which the fractional order system’s disease-free equilibrium points are stable is established. The basic reproduction number is calculated to be ${\mathscr {R}}_{0} = 0.0858 < 1$ and provides important information about the dynamics of the disease in the future. The sensitivity analysis of the integer order model and fractional order model for $\alpha \in (0,1)$ cases are computed. The comparison between the sensitivity analyses of the classical model and the fractional order model shows that the sensitivity analysis of the fractional order model depends on the order of fractional derivatives whereas the classical models do not.

We investigated the fractional optimal control problem by the application of the optimal control theory and used Pontryagin’s Minimum Principle to provide the necessary conditions needed for the existence of its optimal solution. Optimal control interventions involving vaccination of susceptible populations and treatment of infective individuals are incorporated into the fractional order model, The fractional optimal control problem was analyzed theoretically and numerically. The numerical simulation showed that with the help of vaccination and treatment controls over a specified period of time we can eliminate HBV infection.

## Data Availability

The data used are included in the manuscript.
